# Ancient insect vision tuned for flight among rocks and plants underpins natural flower colour diversity

**DOI:** 10.1098/rspb.2023.2018

**Published:** 2023-12-20

**Authors:** Alan Dorin, Mani Shrestha, Jair E. Garcia, Martin Burd, Adrian G. Dyer

**Affiliations:** ^1^ Department of Data Science and AI, Faculty of Information Technology, Monash University, Clayton 3800, Australia; ^2^ Department of Physiology, Faculty of Medicine, Monash University, Clayton 3800, Australia; ^3^ Department of Disturbance Ecology, Bayreuth Center of Ecology and Environmental Research, University of Bayreuth, 95447 Bayreuth, Germany; ^4^ Melbourne Data Analytics Platform, The University of Melbourne, Melbourne Connect, Parkville 3052, Australia; ^5^ Department of Biology, Indiana University Herbarium, Indiana University, Bloomington, IN 47405, USA

**Keywords:** vision, floral colour, insect pollination, chromatic signal, spectral reflectance data

## Abstract

Understanding the origins of flower colour signalling to pollinators is fundamental to evolutionary biology and ecology. Flower colour evolves under pressure from visual systems of pollinators, like birds and insects, to establish global signatures among flowers with similar pollinators. However, an understanding of the ancient origins of this relationship remains elusive. Here, we employ computer simulations to generate artificial flower backgrounds assembled from real material sample spectra of rocks, leaves and dead plant materials, against which to test flowers' visibility to birds and bees. Our results indicate how flower colours differ from their backgrounds in strength, and the distributions of salient reflectance features when perceived by these key pollinators, to reveal the possible origins of their colours. Since Hymenopteran visual perception evolved before flowers, the terrestrial chromatic context for its evolution to facilitate flight and orientation consisted of rocks, leaves, sticks and bark. Flowers exploited these pre-evolved visual capacities of their visitors, in response evolving chromatic features to signal to bees, and differently to birds, against a backdrop of other natural materials. Consequently, it appears that today's flower colours may be an evolutionary response to the vision of diurnal pollinators navigating their world millennia prior to the first flowers.

## 1. Introduction

Unravelling the origins of flower colour signalling to pollinators is fundamental to pollination studies, and to current evolutionary biology and ecology more generally [1]. But naturalists have long been curious about flower structure and colour [2,3]. In the late nineteenth century, Rayleigh [4] had proposed that flower-visiting animals may have different colour vision from humans, a factor anticipated to impact flower coloration if this trait and animal vision are evolutionarily intertwined. This point was later confirmed for prominent flower visitors: bees were found to have phylogenetically conserved trichromatic vision based on UV-, blue- and green-sensitive photoreceptors [5]; birds to have tetrachromatic vision including a long wavelength red photoreceptor class [6]. Humans, by contrast, typically have trichromatic blue, green and red photoreceptors [7].

While biotic and abiotic factors may influence flower coloration, biotic pollination is often the most important [8]. In fact, significant differences in flower coloration have evolved, independent of phylogeny, depending on which animals are a plant's pollinators [9,10]. For example, fly-pollinated flowers are of a yellowish-green appearance to humans [10], while bird-pollinated flowers typically show long wavelength-rich colours that reflect reddish light [9]. Interestingly, fly-pollinated flowers from phylogenetically diverse angiosperms are found within very restricted regions of the colour spaces modelling the perception of both bees and flies [10,11]. In addition, the basal angiosperm stages of flower evolution that have been proposed as mainly fly-pollinated lack ‘bright-coloured’ flowers [12]. These facts suggest that bee and bird pollination are the main drivers of salient flower colouration in modern angiosperms [10,13]. Bird-pollinated flowers' reflectance spectra *marker points*—locations centred on sharp transitions in spectral reflectance known to impact animal vision systems [9,14]—therefore tend to cluster around the longer wavelength region of avian visual sensitivity at approximately 600 nm. By contrast, bees lack a red-sensitive photoreceptor and so most bee-pollinated flowers have spectra with distinctive marker points clustered around 400 nm and 500 nm [14–17]. These spectral properties align closely with psychophysics evidence that bees visually process signals with such features particularly well due to overlap in the photosensitivity of their colour receptors at these wavelengths [5,14,18], while bird colour vision enables sensing of longer wavelengths that bees process less effectively [6,19]. However, although many flower colours may have evolved for bee vision, as we noted, the spectral position of hymenopteran colour photoreceptors predates the emergence of angiosperms and is phylogenetically conserved [5]. Interestingly, it has also been recognized that the spectral positions of these photoreceptors appear to be tuned [20]. We might then wonder, *what did the ancestors of bees look at before flowers appeared on Earth?*

**Figure 1.**
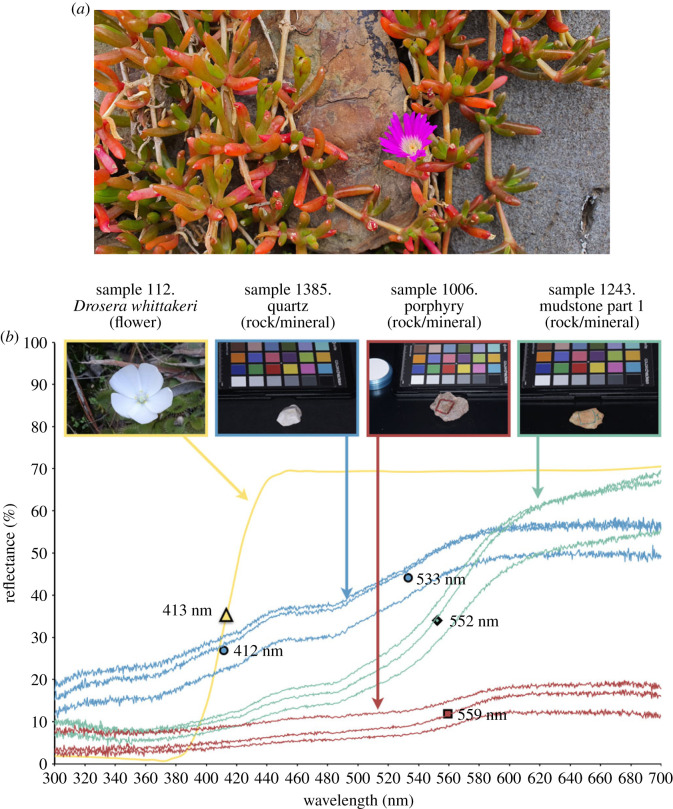
(*a*) Sample surfaces encountered as flower backgrounds by pollinators shown here with Pig face (*Carpobrotus* sp.). Image A.G.D. (*b*) Examples of three sets of three mundane natural surface (raw) reflectance spectra (obtained as described in §2) and a flower spectrum for comparison: sample 1385-Quartz, 1006-Porphyry, 1243-Mudstone, and reading of spectrum for sample 112–*Drosera whittakeri.* 'Mudstone part 1’ measurement refers to it being the first of (in this case) two sections on the heterogeneous material surface whose reflectance was measured. Marker points for the background samples are plotted in the diagram at a 5% threshold on the average reading of the three spectra for each material. The relatively flat profile of the Porphyry (red square marker) contrasts to the intense jump in reflectance of the mudstone (green diamond marker) and the double-stepped rise of the quartz (two blue circle markers) and serves as an example of the variability in the dataset. The flower, included for comparison, has strong UV-absorbing properties but strongly reflects incident visible light across the remainder of the considered spectrum. It exhibits a sudden and rapid reflectance change to generate a marker point at 413 nm. (Image of flower no. 112 in natural surroundings, Atlas of Living Australia obs. 29163827, © Ralph Foster 2019, https://www.inaturalist.org/observations/29163827 used under license: CC-BY-NC 4.0 (Int) http://creativecommons.org/licenses/by-nc/4.0/. Images no. 1006, no. 1243, no. 1385 of samples provided in the dataset are © the authors.).

**Figure 2.**
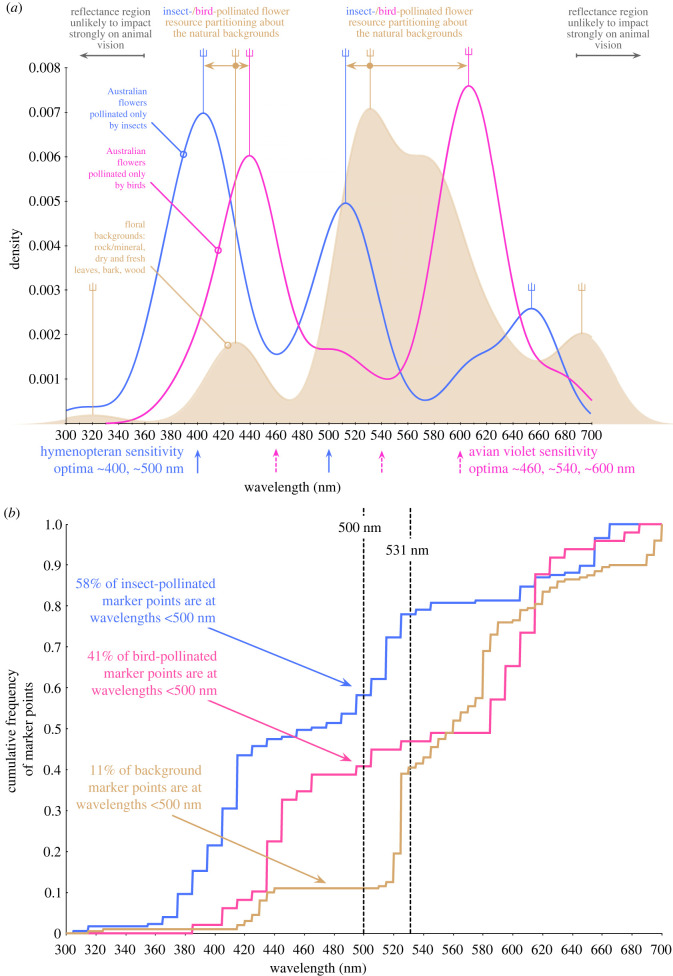
(*a*) Density estimates of the distribution of marker points for simulated backgrounds generated from our database of natural samples (brown, *N* = 507 samples (*n* = 346 rock/mineral samples, 65 green leaves, 96 dry leaves, bark, wood etc.), more than 5% reflectance change threshold), Australian flowers pollinated only by insects (blue, 177 markers, *N* = 153 samples, >20% reflectance change threshold) and pollinated only by birds (pink, 49 markers, *N* = 68 samples, >20% reflectance change threshold). Vertical forked lines indicate modes—the most frequent spectral marker point positions—for each distribution. Bandwidths for kernels were calculated after identifying the number of modes per distribution (electronic supplementary material, table S1): insect modes 404, 513, 654 nm, bird modes 439, 606 nm, background modes 320, 429, 531, 692 nm for 100 000 simulated backgrounds drawn from random sets of 65 green leaves, 65 dry plant materials and 65 rocks and minerals. The figure allows comparison of the positions along the wavelength axis of the strong 20% markers of floral signals that have evolved in response to pressure from the vision systems to which they are signalling, and the 5% markers of simulated backgrounds made from material samples that have not been generated in response to evolutionary selection. Flowers pollinated by violet-sensitive birds have markers peaking to the right of the main simulated background marker peaks, whereas insect-pollinated flowers peak to their left. (*b*) The cumulative frequency of the marker points for simulated Australian natural backgrounds (brown), flowers pollinated only by insects (blue) and birds (pink) reveals that only 11% of background marker points in the simulated assemblages lie to the left of the centre line (500 nm, chosen for illustrative purposes), while 58% of insect and 41% of bird-pollinated flower marker points lie to its left. Collectively, these percentages indicate that the insect-pollinated and bird-pollinated flower markers are both distinct from the backgrounds (having marker points in different regions of the spectrum), and from one another. An alternative reference to the 500 nm centre line for comparing the distribution of insect-/bird-pollinated flowers is suggested by the data—the mode of the simulated background curve (brown), 531 nm. Only 47% of bird-pollinated flowers, but 78% of insect-pollinated flowers lie to its left, highlighting the difference between the flowers.

**Figure 3.**
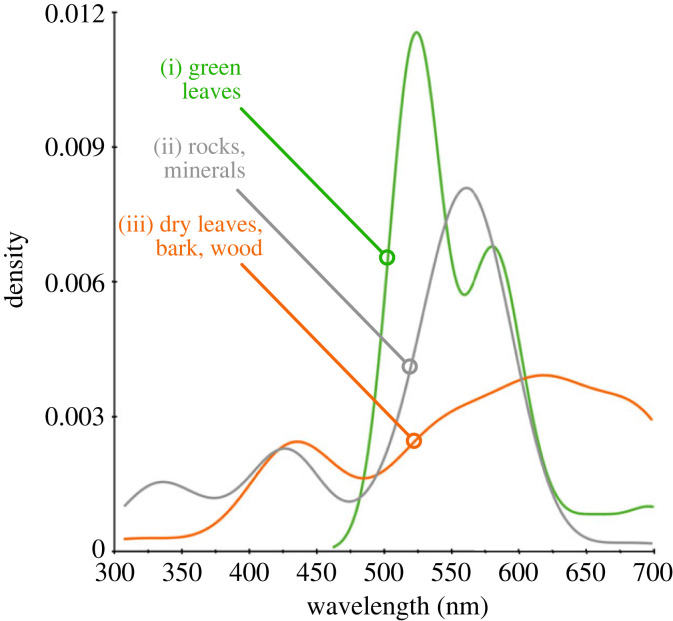
Marker point densities for individual background sample classes (*N* = 507 samples) marker points detected at more than 5% reflectance change over 50 nm interval, bin width = 5 nm (see §2). (i) Green leaves (green line): *n* = 65 samples, 89 marker points, multimodal distribution (*D* = 0.120, *p* < 0.005), modes 524, 580, 696 nm, critical bandwidth = 19.4; (ii) rocks and minerals (grey line): *n* = 346 samples, 246 marker points, multimodal distribution (*D* = 0.052, *p* < 0.001), modes 329, 421, 558 nm, critical bandwidth 26.1; (iii) dry leaves, bark, wood, etc. (orange line): *n* = 96 samples, 129 marker points, multimodal distribution (*D* = 0.062, *p* < 0.001), modes 431, 617 nm, critical bandwidth = 31.9. These individual data are assembled to constitute the overall simulated natural background density shown in figure 2*a* (shaded brown region).

To answer this question we looked to Australia, a geologically ancient continent covering the landscapes of evolutionary history from the Precambrian to the Quaternary [21]. We collected rock and other samples likely to form the pre-floral visual context for animals (e.g. figure 1*a*), across a 2400 km range from the tropical north (16.9°, 145.7°) to the temperate south (39.1°, 146.2°) of the mainland, and used a reflection spectrophotometer [22] to measure their reflectance signatures (figure 1*b*). When we calculated the spectral reflectance curve marker points (figure 1*b*, detail and caption) from these visual background sample data and simulated their assembly into floral backgrounds (see §2), we observed a correspondence between the positions of marker points for the simulated backgrounds and bees’ visual capacities. Specifically, our analysis (§2 and electronic supplementary material, table S1) revealed that spectra assembled into simulated natural backgrounds also have a marker point frequency distribution forming two major peaks within the range expected to influence pollinator vision: one peak (mode = 429 nm, figure 2*a*) is generated by rock, mineral and dead plant samples (figure 3(ii),(iii)); a second major peak (mode = 531 nm, figure 2*a*) is also generated by these materials and spectra of fresh green leaf samples (figure 3(i)). Our analysis also identified minor peaks in the simulated backgrounds (modes = 320 and 692 nm, figure 2*a*; electronic supplementary material, table S1) outside the range where marker points are likely to influence bee pollinator colour discrimination [18].

Hymenopteran insects inhabiting a world before flowers would have been likely to encounter diverse relative abundances of the stimuli we sampled and assembled into simulated natural backgrounds. These stimuli must have provided the information for each insect to have oriented itself [23] by processing spectra with a broad range of markers that constituted its ‘pre-floral’ visual context. This biologically relevant context was set by physical and chemical properties of material light absorption, transmission and reflection that produce colour (e.g. [24]). Unlike floral colour, which has been optimized by natural selection for bee vision [25], background colours were not determined by any evolutionary pressure mediated through organisms' eyes, and therefore they have conveniently remained unchanged over hundreds of millions of years.

Thus, a seemingly plausible theory for the way in which the distribution of flower colours may have arisen is as follows. While flying within the constraints of their limited spatial acuity, bees have to process visual information, such as edges, to orientate and maintain flight stability [23,26]. Arthropods mainly use colour information for discriminating and recognizing objects against the background [27]. These objects may include obstacles on an insect's flight path or camouflaged prey [28]. Similar challenges are likely to be faced by the arthropod ancestor of trichromatic bees. The same set of colour receptors is maintained in modern bees, and so, working from the groundwork laid down by this long-term abiotic–biotic interaction, it appears that some flower species evolved colour signals similar to one another to facilitate visual detection by bees [22].

If it were true that flowering plants have optimized visual signals to improve pollination, then one piece of evidence in support of this would be that their spectral marker points lie on more pronounced reflectance steps than the backgrounds against which they would be perceived, as this would enhance their salience to pollinators' vision systems. To investigate this possibility, we computed the marker points for published flower spectra [9,29] and our background data, centred on a rapid reflectance change (20% or greater reflectance increase within a 50 nm range). The Australian floral data were selected to match the geographical region of our background data, and its colour properties are known to closely match those of large datasets from other regions of the world [13–15]. We found the proportion of flowers with salient markers to be an order of magnitude above that for the individual background samples (electronic supplementary material, table S1), demonstrating evolutionary promotion of salient floral spectra against the unevolved non-floral visual background spectra.

Another piece of evidence that has the potential to support the proposal that flower colours evolved in response to pollinator vision against natural backgrounds relates to the distributions of floral and background spectral marker points. When we subdivided the flower data into two classes according to the flowers’ dominant pollinators within Australia, either tetrachromatic violet-sensitive (VS) birds [6,9] or trichromatic bees, we observed a second major finding (figure 2*a*; electronic supplementary material, table S1). From marker point distributions for the flowers at the salient 20% threshold set against the markers of the simulated backgrounds assembled from our samples at a subtle 5% threshold, we found that bird-pollinated flowers lie in two peaks to the right (modes = 439, 606 nm) of the corresponding marker point frequency peaks for the simulated backgrounds (modes = 429, 531 nm). By contrast, the two major peaks in the frequency distribution of the salient 20% markers for bee-pollinated flowers (major modes = 404, 513 nm) sit left of the corresponding peaks for the simulated backgrounds. This is likely explained by bees' lack of longer wavelength-sensitive photoreceptors and avian pollinators’ superior long wavelength colour vision [9]. The extent of this separation is further demonstrated by the differently skewed cumulative frequency distributions of marker points for the types of flowers and simulated background assemblages (figure 2*b*). For example, only 11% of simulated background marker points lie left of the centre line (500 nm), while 58% of insect- and 41% of bird-pollinated flower marker points lie to its left, indicating that not only are the insect- and bird-pollinated flower marker points differently distributed from the simulated backgrounds, but they are also differently distributed from one another. The difference of the two flower classes from one another is even more apparent if instead we consider the percentages of flower marker points to the left of the main simulated background marker peak mode, 531 nm—78% of insect-pollinated flowers but only 47% of bird-pollinated flowers lie in this region.

The different shifts of the bee- and bird-pollinated flowers' spectral marker point wavelength values about the markers for the simulated backgrounds is consistent with resource partitioning. That is, when feeding from a common source, different animals may self-select resources based on a trait that lowers competition, which in turn promotes the evolution of specialization [30]. The key that appears to unlock this ancient evolutionary process is the need of flying pollinators, especially bees, to have first orientated themselves in a complex environment. Thus, rocks, soil, sticks, bark and leaves plausibly set the stage for flower colour hundreds of millions of years ago, even before the first buds bloomed.

## 2. Methods

### (a) Material samples, data collection

Background materials data (non-floral surfaces): rocks, minerals, sand, shells, dry leaves, dry bark, wood, dry seeds, green leaves.

• Data are available at Dryad: doi:10.5061/dryad.fn2z34v2c.
• Samples were collected across a 2400 km range from the tropical north (16.9°, 145.7°) to the temperate southern tip (39.1°, 146.2°) of mainland Australia.
• *N* = 507 total natural background surface data samples were collected: *n* = 65 green leaves; *n* = 96 dry leaves, bark, wood, etc., samples; *n* = 346 rocks/mineral samples.

Flower data (petal surfaces): insect- and bird-pollinated flowers.

• Data available at Dryad: doi:10.5061/dryad.fn2z34v2c.
• Data have previously been described and analysed [9,29].

### (b) Spectral reflectance measurement

Reflectance curves were measured for background material and floral samples from 300 to 700 nm (see §2c below) using a spectrophotometer with quartz optics and a PX-2 pulsed xenon light source (USB2000+, Ocean Optics Inc., Dunedin, FL, USA) attached to a computer running SPECTRA SUITE software. Reflectance profiles were measured relative to a Lambertian PTF WS-1 reflectance standard (Ocean Optics, USA). Multiple readings, usually three, were taken from within a square region of each surface (see figure 1*b* for examples). Where sample surfaces were conspicuously heterogeneous to human visual systems, several sections were sampled individually and used in this study. In the case of fresh green leaves specifically, one measurement was taken near the base, one in the middle and one near the tip of each. These three spectra were then used to calculate an average reflectance spectrum for the leaf.

To assist us in making accurate spectrophotometer readings, we built a black, curved-wall sample enclosure and covered this with a black cardboard lid. The lid was perforated with a tiny hole through which a fibre-optic light was channelled. This prevented ambient light from hitting the sample during measurement. Room lighting was turned off. The optical fibre was held in an aluminium block that also helped to eliminate stray illumination. The fibre was held approximately 6 mm above each sample, illuminating a circular patch of 3–4 mm in diameter. Sample data points recorded between 300 and 700 nm in wavelength that represent reflectance spectra for three background surfaces are illustrated in figure 1*b*.

### (c) Marker point calculations

Marker points are locations on surface spectral reflectance curves that are centrally located within sudden changes in spectral reflectance (figure 1*b*) [14,31]. The severity of the jump is measured as a threshold over which it occurs from its base reflectance to its peak. For this study, marker points were identified as the midpoint of any change in reflectance of at least a pre-specified percentage threshold value, occurring within a wavelength range of less than 50 nm. We calculated thresholds for each background sample at 5% reflectance jump and for each flower sample at 20% reflectance jump, in order to compare the properties of different materials. The 20% threshold for flower spectra was taken from the literature as being suited to such evolutionarily enhanced signals [16]. The 5% threshold for natural backgrounds that do not constitute evolutionarily enhanced reflectance curves was determined empirically to provide a similar mean number of marker points per sample to that given by the 20% threshold for the flowers (see electronic supplementary material, table S1).

Marker point calculations were performed using the Open Source Spectral-MP software [31] with parameters: threshold 5% (backgrounds), 20% (flowers); range 50 nm; smoothing window 21 points; lookahead 5 points; interval 300–700 nm. The study interval of 300–700 nm was selected to ensure that we analysed regions in which the spectrophotometer readings were reliable, noting that the extremities of these regions are outside the range of sensitivity of avian and hymenopteran pollinator vision (figure 1*b*).

### (d) Simulation of natural background visual characteristics

From the *N* = 507 natural background samples, we generated 100 000 subsets of randomly chosen samples *without* replacement, each comprising (i) 65 green leaf samples (this sub-group, being the group with the lowest number of samples, acted as the limiter on group size), (ii) 65 dry leaf, bark, wood, etc., samples and (iii) 65 rock/mineral samples. From each of these 100 000 subsets, we resampled *with* replacement from its collection of (3 × 65 = ) 195 elements to simulate a random background such as might be viewed behind a flower perceived by a pollinator. In this way, a simulated random background may include, for instance, duplicate samples and/or unequal numbers of elements drawn from each sub-group (i), (ii) or (iii). Marker points for all of the samples in each simulated random background were then binned at 5 nm steps from 300 to 700 nm (e.g. a marker point located at 426.11 nm was rounded to 425 nm) using Python Pandas' cut function (see https://pandas.pydata.org). We selected this bin size based on empirical psychophysics experiments to determine hue discrimination functions (see von Helversen [18] on bees and Wright [32] on birds). The bin size is not so small as to require discriminatory abilities above what has been experimentally determined, nor so large that vision systems typical of pollinators could reliably distinguish stimuli within a bin. The markers for all 100 000 iterations of this procedure were analysed to generate the statistics and figures we report.

### (e) Statistical tests for modality of the data

We initially tested the null hypothesis of unimodality using a Dip test [33], which tests for multimodality by calculating the maximum difference between the empirical and unimodal distribution, minimizing such a difference. This method is more robust than bandwidth-based methods such as Silverman's [34] as the Dip test does not require input of the unknown magnitude of the kernel bandwidth.

Following the rejection of the null hypothesis of unimodality for the three distributions (green leaves; rocks and minerals; dry leaves, bark, wood), the positions of the modes were located using the function *locmode* available in the multimode package for R [35]. The results of these analyses are presented in figure 3.

Our simulation of natural backgrounds generated by subsampling from the natural background classes above (see §2d, above) resulted in a median number of 200 marker points (68–488, 95% CI) for a simulated natural background within the spectral range of 300–700 nm and where markers were binned at 5 nm intervals. Analysis results are provided in electronic supplementary material, table S1 and figure 2*a* brown curve.

## Data Availability

Background surface data are available online at Dryad Digital Repository: http://dx.doi.org/10.5061/dryad.fn2z34v2c [36]. Flower data have previously been described and analysed [9,28] and are available at Dryad Digital Repository: http://dx.doi.org/10.5061/dryad.fn2z34v2c [36]. Marker point analysis software is available online [31].
